# Potential Utilization of End-of-Life Vehicle Carpet Waste in Subfloor Mortars: Incorporation into Portland Cement Matrices

**DOI:** 10.3390/ma18153680

**Published:** 2025-08-05

**Authors:** Núbia dos Santos Coimbra, Ângela de Moura Ferreira Danilevicz, Daniel Tregnago Pagnussat, Thiago Gonçalves Fernandes

**Affiliations:** 1 Postgraduate Program in Civil Engineering: Construction and Infrastructure, Federal University of Rio Grande do Sul, Porto Alegre 90040-000, Brazil; angela.danilevicz@ufrgs.br (Â.d.M.F.D.); daniel.pagnussat@ufrgs.br (D.T.P.); 2 Liberato Salzano Vieira da Cunha Technical School Foundation, Novo Hamburgo 93340-140, Brazil; thiago.fernandes@liberato.com.br; 3 Rio Grande do Sul State Department of Traffic (DETRAN/RS), Porto Alegre 90030-010, Brazil

**Keywords:** end-of-life vehicle, automotive carpet waste, subfloor mortar, construction materials

## Abstract

The growing need to improve the management of end-of-life vehicle (ELV) waste and mitigate its environmental impact is a global concern. One promising approach to enhancing the recyclability of these vehicles is leveraging synergies between the automotive and construction industries as part of a circular economy strategy. In this context, ELV waste emerges as a valuable source of secondary raw materials, enabling the development of sustainable innovations that capitalize on its physical and mechanical properties. This paper aims to develop and evaluate construction industry composites incorporating waste from ELV carpets, with a focus on maintaining or enhancing performance compared to conventional materials. To achieve this, an experimental program was designed to assess cementitious composites, specifically subfloor mortars, incorporating automotive carpet waste (ACW). The results demonstrate that, beyond the physical and mechanical properties of the developed composites, the dynamic stiffness significantly improved across all tested waste incorporation levels. This finding highlights the potential of these composites as an alternative material for impact noise insulation in flooring systems. From an academic perspective, this research advances knowledge on the application of ACW in cement-based composites for construction. In terms of managerial contributions, two key market opportunities emerge: (1) the commercial exploitation of composites produced with ELV carpet waste and (2) the development of a network of environmental service providers to ensure a stable waste supply chain for innovative and sustainable products. Both strategies contribute to reducing landfill disposal and mitigating the environmental impact of ELV waste, reinforcing the principles of the circular economy.

## 1. Introduction

Improving the management of end-of-life vehicle (ELV) waste and mitigating its environmental impacts are critical and increasingly global challenges [1,2,3]. As long as the automotive industry fails to prioritize the integration of sustainability principles and zero-waste strategies in the design and development of vehicles and composite materials, the negative consequences of inadequate or informal ELV processing are likely to escalate. Beyond environmental harm, such practices also lead to the loss of valuable resources, given that ELVs are significant sources of secondary raw materials, comprising various components with high potential for reuse and recycling [4,5,6].

Simultaneously, the construction industry has been striving to reduce the environmental impacts of its activities by adopting cleaner technologies, particularly through the recycling and upcycling of waste materials. In this context, there is a growing emphasis on product sustainability, reflected in the increasing use of alternative materials to partially or fully replace Portland cement and natural aggregates [7,8,9]. These strategies not only decrease the consumption of non-renewable natural resources but also help to reduce greenhouse gas emissions associated with cement manufacturing and aggregate extraction, thus aligning with the principles of the circular economy [3,10,11,12,13].

Given this scenario, numerous initiatives have been explored to increase recycling rates and promote environmentally sound disposal of ELVs, particularly targeting their application in civil construction [1]. In the studies conducted by Wong et al. [5], Handoko et al. [14], and Go et al. [15], the recycling of metallic waste—primarily originating from monoblocks, sheet metal, wheels, engines, and transmissions and composed mainly of iron, aluminum, and copper—resulted in raw materials to produce profiles, sheets, and reinforcing bars for construction use. Polymers derived from ELVs were examined by Liu et al. [16] and Wong et al. [5], who evaluated their reuse in thermoacoustic partition panels, asphalt mixtures, and carpets and as aggregates in cementitious composites. Additionally, the incorporation of shredded tires as partial aggregate replacements in cement-based materials was investigated by Rashad [17], Ataria and Wang [18], and Khaloo et al. [19], while Eusuf and Al Hasan [20] assessed the use of rubber tire strips as roofing coatings. Yu et al. [21] further advanced this field by developing ultra-high-performance concrete reinforced with recycled tire steel fibers, analyzing its mechanical performance and crack resistance. The reuse of ELV-derived glass waste has also been studied in the production of asphalt, roofing tiles, and thermal and acoustic insulation panels and as a substitute for conventional aggregates in concrete and ceramic composites [5,15].

It is essential to note that waste generated from ELVs encompasses materials beyond those commonly recovered. The dismantling process generates a wide range of additional residues that require environmentally responsible management strategies, including appropriate treatment, reuse, or recycling. Among these, textile materials stand out due to their extensive presence in vehicles, with over 40 distinct applications covering a combined surface area of more than 35 m^2^, as reported by Innovation in Textile [22]. However, only about 10% of this total (approximately 3.5 m^2^) corresponds to visible textiles, primarily carpets located in the passenger compartment and trunk [23]. Additionally, driven by increasing demands for comfort, acoustic insulation, and safety, the use of automotive textiles has grown substantially in recent decades—from around 20 kg per vehicle in the early 2000s to approximately 35 kg in current models [22].

According to data presented by Coimbra [24], between 2011 and 2020, approximately 340,000 vehicles were recycled in the state of Rio Grande do Sul, Brazil. This volume is estimated to have generated approximately 1.9 million m^2^ of automotive carpet waste (ACW), the vast majority of which was ultimately sent to landfills. On an annual basis, this corresponds to approximately 190,000 m^2^ of ACW, which, despite its low economic value and limited commercial demand, exhibits a high volume-to-mass ratio due to its low apparent density. This characteristic presents logistical and environmental challenges for storage and final disposal, thereby highlighting the need for technologically viable and environmentally sustainable strategies for its recovery and reuse.

In this context, the study conducted by Lesiak et al. [25] is particularly noteworthy. The authors developed a polymer composite based on low-density polyethylene reinforced with shredded automotive carpet waste, demonstrating its potential for use in anti-erosion mats, geotextile composites for landscaping, corrugated membranes, and waterproofing systems. Complementarily, Wong et al. [5] highlighted the thermal and acoustic properties of automotive textile waste, reinforcing its suitability for developing innovative construction materials. Despite these advances, a significant gap remains in the literature regarding the use of automotive textile residues—particularly carpets—in civil construction applications.

Therefore, it is essential to develop strategies that promote the valorization and environmentally responsible disposal of ACW by incorporating it into mortar-based cementitious matrices for use in the construction industry. Such applications play a significant role in mitigating the environmental impacts associated with waste disposal and the extraction of virgin raw materials. Moreover, they generate added value from economic, environmental, and social standpoints by fostering the development of innovative construction solutions. Simultaneously, these initiatives strengthen the value chain of environmental service providers and enhance the recovery of automotive materials, aligning with the core principles of the circular economy.

This study aimed to develop and assess cementitious composites for civil construction by incorporating end-of-life vehicle carpet waste into subfloor mortars. The primary focus was on maintaining or improving performance concerning conventional reference materials while proposing innovative and sustainable alternatives for impact noise insulation in flooring systems. An experimental program was conducted in which mortar composites were produced with different contents of ACW (2%, 5%, 10%, 15%, and 20%). In addition to their physical and mechanical characterization, the composites were evaluated for dynamic stiffness, showing consistent and significant improvements across all incorporation levels. These findings highlight the potential of ACW-based composites as viable materials for applications that demand acoustic performance, aligning with sustainability and circular economy principles.

## 2. Materials and Methods

To conduct this research, subfloor mortars were produced with the incorporation of ACW at different percentages relative to the cement mass. An experimental program was designed and divided into three phases. First, a physical characterization of the materials used in the composites was carried out. Next, the sample preparation method was defined and presented. Finally, in the third phase, the properties of the samples were evaluated through standardized tests to determine the composition that provides the best performance for the produced composites.

### 2.1. Material Characterization

The cement selected for this study was Portland Cement CP V-ARI (similar to ASTM type III cement), which complies with the requirements of NBR 16697 [26] and has a specific gravity of 3120 kg/m^3^. This cement is characterized by higher purity compared to other commercial Brazilian types of cement as it does not contain pozzolanic admixtures.

**Figure 1:**
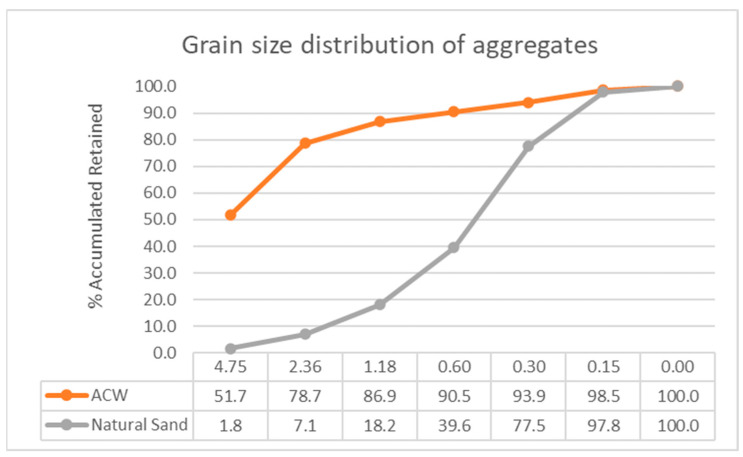
Granulometric distribution of aggregates.

Natural sand was used as the fine aggregate in this research. Its particle size distribution was determined and is presented in Figure 1. All the sand required for the experimental work was dried in an oven until a constant mass was achieved and then stored in sealed containers to prevent moisture absorption.

The ACW was kindly provided by companies specializing in vehicle dismantling and recycling in the cities of Novo Hamburgo and Canoas, Brazil, in support of this research. During the ELV carpet waste selection process, four types were identified. They had different stiffness, layers, and weights per square meter. Before characterizing the sample, a mixed blend was created to better reflect the operational reality of environmental service providers, who typically handle all carpet types together without prior separation.

**Figure 2:**
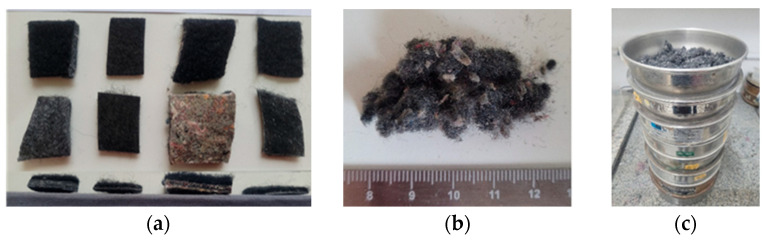
ACW: (**a**) carpet types, (**b**) processed, and (**c**) sieves.

Once collected, the material was transported to a Ronaplast Plastics Industry and Commerce Ltd., Vale Real, Brazil (polymer recycling company) for processing as it was necessary to grind the waste to reduce particle size and improve its incorporation into the composites. For this purpose, an MGHS300A mill manufactured by the Brazilian company SEIBT (Nova Petrópolis, Brazil) was used. The machine features rotating flanges that shear the material, which is then passed through a 10 mm sieve to ensure uniformity in the resulting fiber size (Figure 2).

In the laboratory, granulometric analysis of the ACW obtained from the beneficiation process was conducted to determine particle sizes and their respective proportions, thereby obtaining the grain size distribution in accordance with NBR 17054 [27]. Figure 2b shows the residue resulting from the beneficiation process, while Figure 2c displays the sieves used in the granulometric test.

### 2.2. Sample Preparation

**Figure 3:**
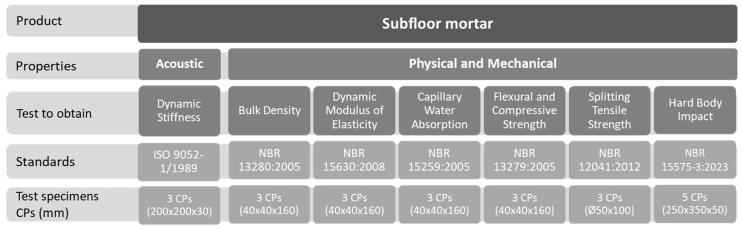
Detailed experimental program.

The samples were produced in accordance with current Brazilian standards to evaluate the performance of the developed composites. Figure 3 outlines the experimental program, detailing the properties assessed, tests conducted, relevant standards, and the number and size (millimeters) of specimens required for each evaluation.

**Figure 4:**
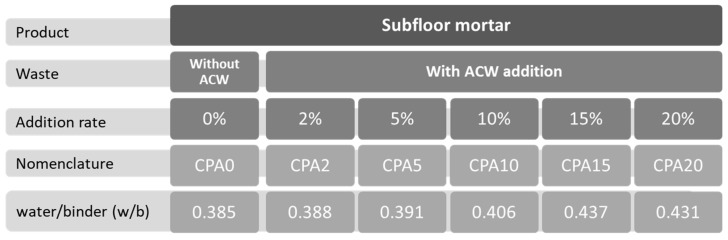
Percentages and nomenclature of subfloor mortar samples.

The test specimens were prepared using dry-consistency subfloor mortar with the addition of ACW by cement mass at five different percentages: 2%, 5%, 10%, 15%, and 20%. The reference sample contained no ACW (0%), and the percentages of 2% to 20% were defined based on previous laboratory studies, where it was noted that levels above 20% produced dry mortars without adequate workability, probably compromising their use in practical situations. Figure 4 presents the six developed mixes, their associated nomenclature, and the water/binder (w/b) ratio used in the preparation of each sample. For mortar production, a volume ratio of 1:3 cement to sand was adopted, following the approach of Führ et al. [28], who emphasized the importance of a cement-rich mix for testing.

After establishing the w/b ratio for the reference mortar mix, the same manual procedure was applied to determine the appropriate ratio for each composite mix. Consequently, each mix was prepared separately using the mortar mixer to ensure the correct w/b ratio for each percentage of ACW added. As the percentage of waste increased, so did the volume of water absorbed since the shape and texture of the waste directly affect the workability of the mixtures [29,30,31].

After preparing the mortar, the test specimens (CPs) were molded following ISO 9052-1 [32], NBR 13279 [33], and NBR 12041 [34] standards. The mortars were compacted and left to cure naturally for 48 h before demolding. Each batch produced in the mortar mixer yielded enough material to fabricate all specimens required for the characterization tests. The specimens were then identified, weighed, and stored in a humid chamber until testing.

### 2.3. Property Evaluation

The physicochemical properties of the ACW were conducted following standardized tests and regulations, including Fourier Transform Infrared (FTIR) Spectrometry (ASTM E1252) [35], DSC—Differential Scanning Calorimetry (ASTM D3418) [36], TGA—Thermogravimetric Analysis (ASTM E1131:2020) [37], Optical Microscopy, and Scanning Electron Microscopy.

**Figure 5:**
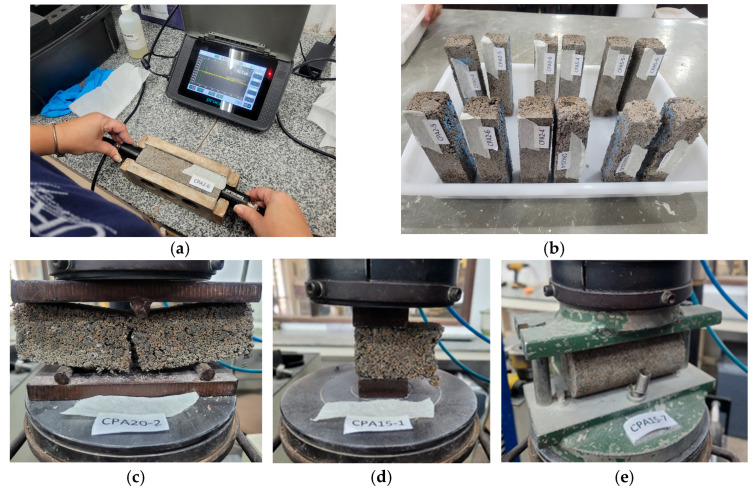
Tests performed: (**a**) dynamic modulus of elasticity, (**b**) water absorption by capillarity, (**c**) tensile strength in bending, (**d**) axial compressive strength, and (**e**) tensile strength by diametral compression.

The physical and mechanical properties of the mortar composites with ACW were determined following the standards specified in Figure 3. The composites were tested after 28 days of curing, except for the water absorption test, which was performed after 50 days, and the dynamic stiffness test, conducted after 180 days. The following tests were performed: bulk density (NBR 13280) [38], dynamic modulus of elasticity (NBR 15630) [39], capillary water absorption (NBR 15259) [40], flexural tensile strength, axial compressive strength (NBR 13279) [33], splitting tensile strength (NBR 12041) [34], hard body impact resistance (NBR 15575-3) [41], and dynamic stiffness (ISO 9052-1) [32]. Some of them are shown in Figure 5.

The dynamic stiffness test was conducted by ISO 9052-1 [32], using three samples per ACW percentage. Each sample measured 200 mm × 200 mm × 30 mm and was cured for 180 days. Prior to testing, the samples were covered with a waterproof plastic film and coated with a 5 mm layer of gypsum plaster to level the surface. The loading plate was applied for 10 s, while the plaster was still wet, after which the samples were allowed to cure for 24 h to ensure complete drying.

**Figure 6:**
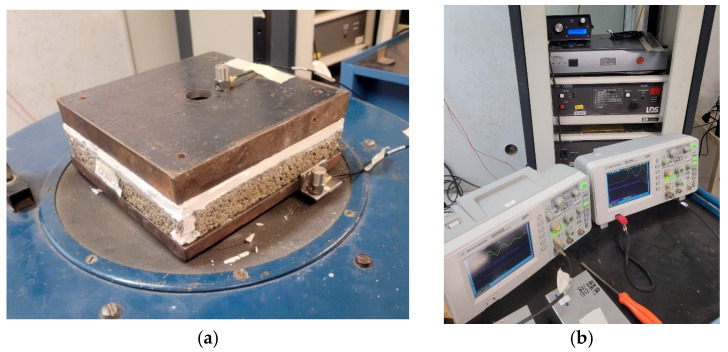
Dynamic stiffness tests: (**a**) sample mounted for testing; (**b**) signal generator, amplifier, and oscilloscopes used for scanning resonance frequencies.

After drying, each sample was placed on a clamping device connected to the vibration equipment, with an 8 kg metal plate positioned on top. The test utilized a vertical shaker vibrator (Thüringer Industriewerk Rauenstein—Schalkau, Germany), capable of operating across various frequencies, speeds, and acceleration ranges and accommodating samples weighing up to 50 kg. Two Brüel & Kjær accelerometers (Nærum, Denmark) were attached to the setup to measure accelerations caused by changes in the vibrator’s frequency, with data recorded via oscilloscopes (Figure 6a). A signal generator and amplifier controlled the frequency sweep during the test (Figure 6b).

The resonance frequencies were determined by exciting the assembly and observing the sinusoidal waveforms on the oscilloscope, with a vertical vibration amplitude of 0.2 G, as described by Borges [30]. The fundamental frequency corresponded to the highest amplitude observed during the frequency sweep. Three specimens were tested for each composite composition (0% to 20% ACW), and the average resonance frequencies were used to calculate the apparent dynamic stiffness, s′_t_ [MN/m^3^], following Equation (1) of ISO 9052-1 [32].

s′_t_ = 4 · π^2^ · m′_t_ · f_r_^2^ (1)

where f_r_ is the resonance frequency of the system [Hz], and m′_t_ is the total mass per unit area applied to the tested sample [kg/m^2^].

Additionally, the resonance frequency and dynamic stiffness values were used to estimate the impact sound reduction of floating floors, following the ISO 12354-2 [42] standard, as calculated by Equation (2).

∆L = 30 · log (f/f_0_) (2)

where ∆L is the reduction in impact sound pressure level [dB], f is the central frequency of the octave band [Hz], adopted as 500 Hz according to Schiavi et al. [43], and f_0_ is the resonance frequency calculated based on the surface mass of the floating floor system (mortar/ACW composite). The results obtained from the tests were statistically analyzed using Analysis of Variance (ANOVA).

## 3. Results and Discussion

### 3.1. Characterization of Automotive Carpet

**Figure 7:**
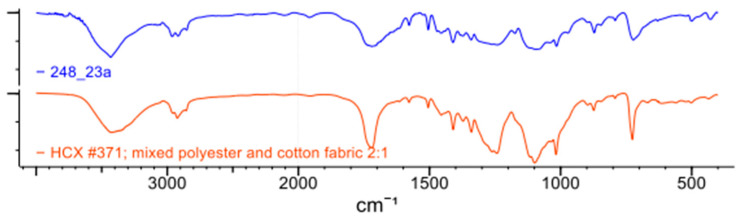
FTIR spectrum of the ACW sample compared to the Wiley Science Solutions database, highlighting characteristic bands corresponding to PET and polyester/cotton blends.

**Figure 8:**
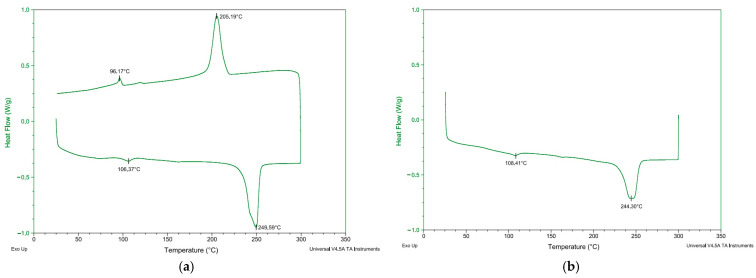
Differential Scanning Calorimetry (DSC) curves for the ACW sample: (**a**) first heating and cooling cycle; (**b**) second heating cycle.

The analyses performed on ACW indicated that the material consists of a polymeric blend, predominantly polyethylene terephthalate (PET)-based. FTIR spectroscopy (Spectrum Two FT-IR—Perkin Elmer, Shelton, CT, USA) identified characteristic bands of PET, with spectral features similar to a polyester (PES) and cotton mixture, based on comparisons with the Wiley Science Solutions database (Figure 7). Differential Scanning Calorimetry (DSC Q20 -TA Instruments) revealed endothermic melting peaks between 244 °C and 250 °C, along with an exothermic crystallization peak at 205 °C, consistent with the thermal behavior of thermoplastics such as PET. Additionally, minor peaks associated with polyethylene (PE) family polymers were observed (Figure 8a,b). Thermogravimetric Analysis (TA Instruments TGA Q50—New Castle, DE, USA) indicated a single mass loss event occurring between 370 °C and 515 °C, with the maximum degradation rate at 450 °C. A residual mass of 16% remained, attributed to unidentified components.

**Figure 9:**
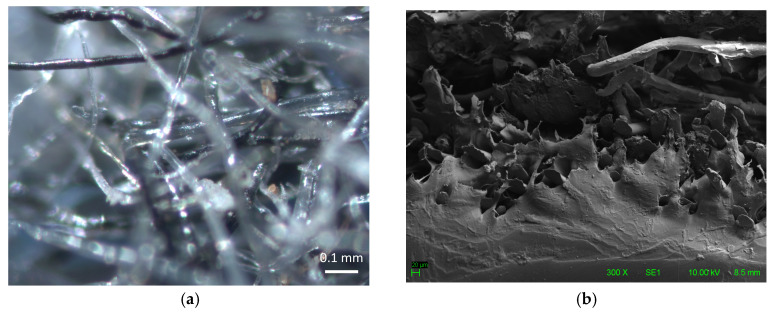
ACW microstructural analysis: (**a**) polymeric fibers observed under stereomicroscope (OM, 100×); (**b**) fibers adhered to the binder, observed under scanning electron microscope (SEM, 300×).

In addition, the microstructure of the ACW was analyzed using a stereomicroscope (OM) at 100× magnification (ZEISS Stemi 508—Carl Zeiss Microscopy, Jena, Germany), and a scanning electron microscope (SEM) at 300× magnification (Hitachi—TM3000, Tokyo, Japan). This microstructural analysis enabled a detailed characterization of the residue, allowing the observation of fibers and agglomerates (Figure 9a) and their adhesion to the binder matrix (Figure 9b), as well as their morphological compatibility with typical textile materials associated with polymers.

### 3.2. Cementitious Composites with ACW: Characterization and Analysis

The loaded surfaces of the test specimens were properly prepared and leveled prior to testing in accordance with the procedures recommended by the applicable technical standards. This step aimed to ensure uniform contact with the testing equipment, minimizing potential interference with the results. Despite the careful preparation, the observed standard deviation values may be attributed to the inherent heterogeneity of the developed composite, particularly due to the incorporation of fibrous residues, which tend to exhibit a non-uniform distribution within the cementitious matrix. Nevertheless, the deviations were analyzed and considered acceptable within the experimental context. The results of the mortar characterization tests are summarized in Table 1. These results were analyzed using Analysis of Variance (ANOVA) with the Statistica 7.0 software, considering a 95% confidence level, in order to assess the significance of the individual variables and their interactions; these results are presented in Table 2.

**Figure 10:**
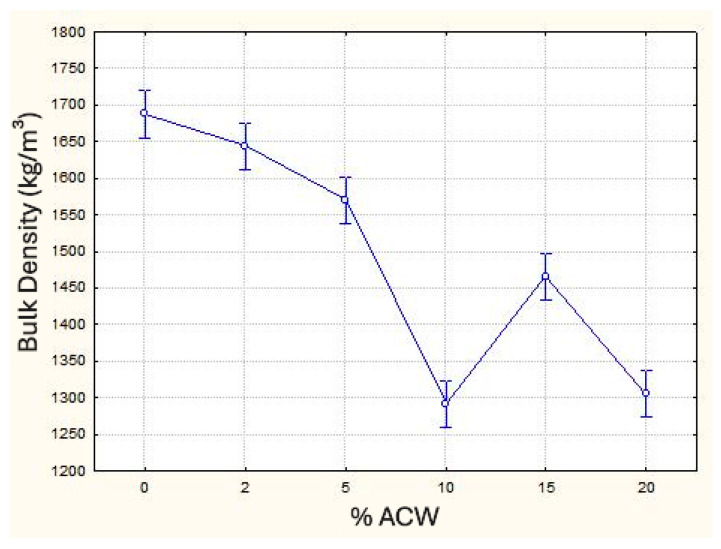
Variation of mass density (kg/m^3^) according to the percentage of ACW.

Table 1 shows that incorporating ACW into the mortar resulted in a reduction in mass density, with decreases of up to 23%, depending on the amount of waste added. This reduction is attributed to the lower density of the ACW, which leads to an increase in the composite’s porosity (Figure 10). While this increase in porosity can negatively affect mechanical performance, it may enhance acoustic properties. The presence of fibrous or porous materials within the voids of the composite can contribute positively to reducing impact noise, as supported by Brancher et al. [44] and Borges et al. [45]. The ANOVA results (Table 2) confirmed that ACW incorporation has a statistically significant effect on reducing bulk density. Additionally, the mean comparison test (Duncan) indicated no significant differences between the 0% and 2% ACW samples, as well as between the 10% and 20% ACW samples, as illustrated in Figure 10.

**Figure 11:**
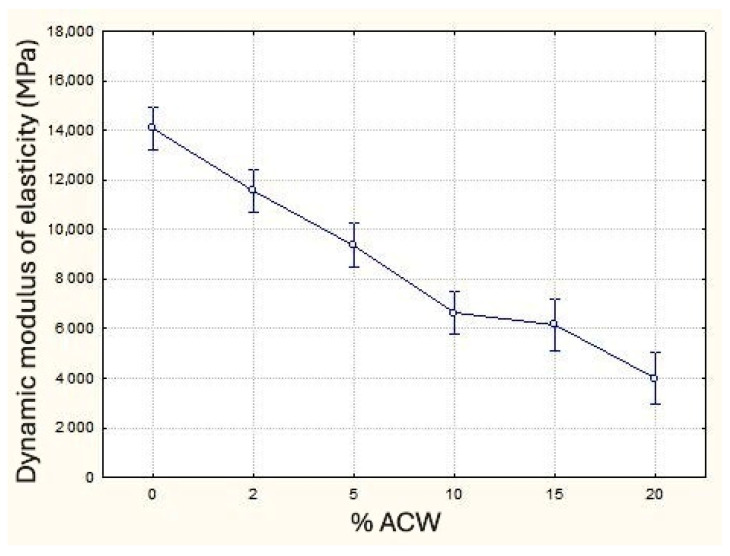
Dynamic modulus of elasticity (MPa) as a function of ACW content (%).

Regarding the dynamic modulus of elasticity, the results indicate that increasing the incorporation of ACW into mortars leads to a reduction in this property. That is primarily attributed to the decrease in mass density and the consequent increase in porosity. This trend aligns with findings from previous studies by Ahmed et al. [46], Führ et al. [28], and Siddique et al. [47], who also investigated the incorporation of various types of waste into cementitious composites. Although this reduction negatively impacts the stiffness of the composite, it may provide benefits in terms of acoustic performance [44] and contribute to a reduction in crack formation in subfloor mortars [48]. The Duncan multiple range test revealed that only the 10% and 15% ACW samples do not show a statistically significant difference between them, whereas all other groups are statistically distinct. Figure 11 illustrates the behavior of this property as a function of ACW content.

**Figure 12:**
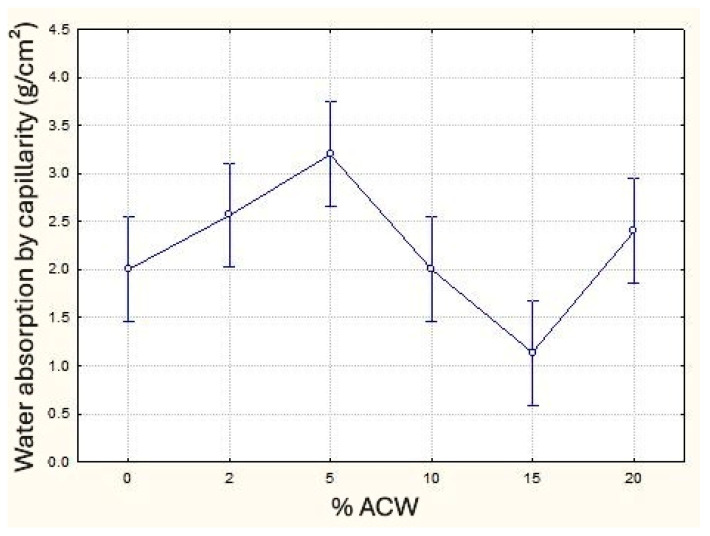
Water absorption by capillarity (g/cm^2^) as a function of ACW content (%).

A clear pattern of capillary water absorption behavior could not be identified. Further investigations into the pore structure are required to definitively determine how ACW fibers influence this property, either beneficially or detrimentally. In the capillary water absorption test, an increase in absorption was expected with higher levels of incorporated ACW due to the greater porosity caused by these additions, as highlighted by Fashandi et al. [49], who noted that water absorption of carpet waste is primarily a physical phenomenon. However, this increase was only observed up to 5% ACW incorporation, as shown in Figure 12. At 10% and 15% ACW, absorption decreased, followed by an increase again at 20%, this last percentage closer to that described by Asasutjarit et al. [50]. Further testing with ACW levels exceeding 20% would be necessary to confirm the observed absorption trend. Analysis of means indicated that the 15% ACW sample differs statistically from all others, while the 5% ACW sample shows significant differences compared to both the reference (0%) and the 10% samples.

**Figure 13:**
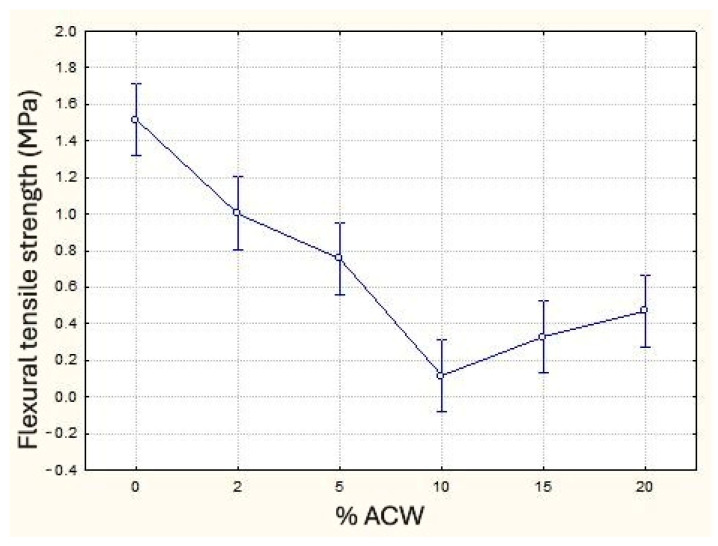
Flexural tensile strength (MPa) as a function of ACW content (%).

The results of the flexural tensile strength tests showed that the incorporation of ACW significantly reduced this property, with reductions reaching up to 92% at 10% ACW content, depending on the percentage added (Figure 13). The decrease in bulk density and dynamic modulus are consistent with the decrease in flexural tensile strength at ACW contents up to 10%. At 15% and 20% contents, this behavior was different, with a slight increase in flexural tensile strength. It is believed that the greater fiber content in these samples may contribute to traction gains, compensating for the lower bulk density, as reported in studies by Borges [30], Fashandi et al. [49], Führ et al. [28], and Olmeda et al. [51]. Further studies are needed to determine whether higher percentages would maintain this behavior, although, as previously mentioned, previous studies found that contents above 20% produced mortars with inadequate workability and poor moldability. This trend, similar to the pattern observed in capillary absorption, hints at a possible shift in behavior, as also noted by Rossignolo [31]. Analysis of means revealed no statistically significant difference between the 2% and 5% samples, between 10% and 15%, and between 15% and 20%, while other group comparisons showed significant differences.

**Figure 14:**
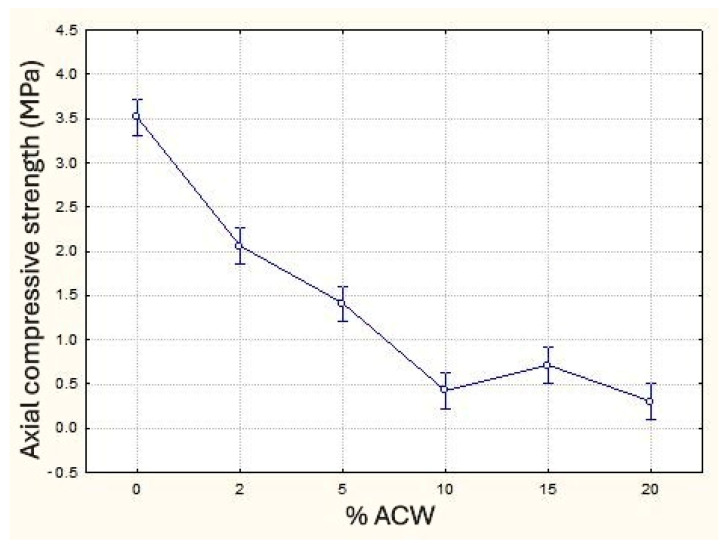
Axial compressive strength (MPa) as a function of ACW content (%).

The axial compressive strength decreased with increasing carpet waste content (Figure 14), exhibiting a trend similar to that observed for flexural tensile strength, as reported in studies by Awal and Mohammadhosseini [52], Borges [30], Fashandi et al. [49], Führ et al. [28], and Tutikian et al. [53]. Reductions of up to 88% were observed at 10% ACW incorporation. Interestingly, the sample with 15% ACW showed a smaller reduction (80%) compared to the 10% sample, while the 20% ACW sample exhibited a more pronounced decrease (91%). Due to its less fluid consistency—a characteristic of subfloor mortars commonly used in Brazil—the inclusion of ACW led to reduced plasticity and moldability. This, in turn, resulted in an increase in internal voids and a decrease in bulk density, which may explain the significantly lower compressive strength values observed. This behavior suggests a potential change in strength characteristics at higher ACW contents, warranting further investigation. Statistical analysis of means revealed no significant difference among the 10%, 15%, and 20% samples, whereas all other samples differed significantly from one another.

**Figure 15:**
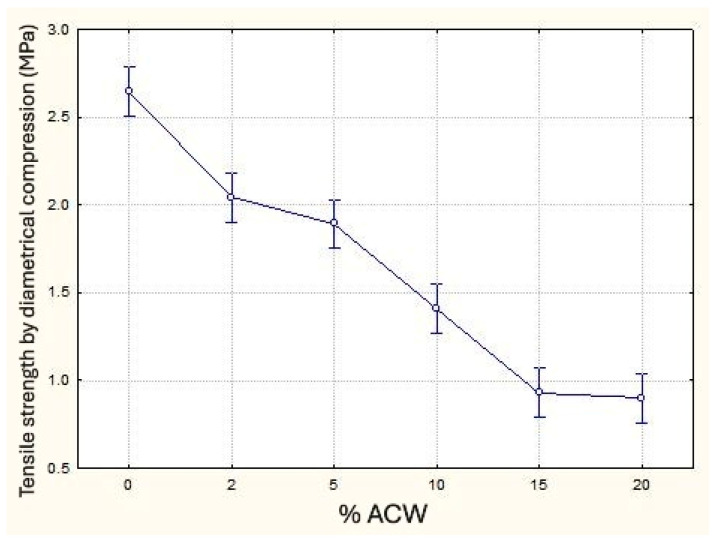
Tensile strength by diametrical compression (MPa) as a function of ACW content (%).

As observed for other mechanical properties, the tensile strength under diametral compression decreased as the ACW content increased (Figure 15), reaching reductions of up to 66% compared to the reference sample. The low compressive strength compared to the expected correlation with flexural tensile values can be explained—albeit in an initial and exploratory context—by the behavior of the ACW residue as a fiber incorporated into the cementitious matrix. Similar to other fibers commonly integrated into mortars, ACW seems to exert a more pronounced beneficial effect on tensile properties rather than compressive strength, akin to the performance of commercially used fibers for similar applications. Statistical analysis of means revealed that the samples containing 2% and 5% ACW, as well as those with 15% and 20% ACW, did not exhibit statistically significant differences between them. In contrast, all other ACW percentages showed significant differences.

After performing the hard body impact tests, a visual inspection was conducted at the points of impact to assess possible damage, such as cracks, fissures, or ruptures. Impacts with a steel sphere weighing 0.5 kg (smaller dimension) did not cause significant damage to any of the samples.

When subjected to an impact with a 1 kg sphere (larger dimension) at an energy of 10 Joules, all samples presented only superficial marks, with no occurrence of cracks, fissures, or structural failures, regardless of the ACW content.

**Figure 16:**
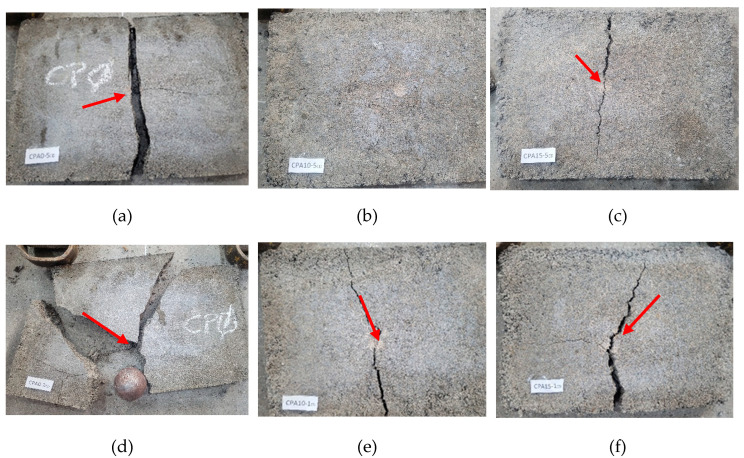
Hard body impact test results: damage observed after impact with 20 Joules: (**a**) 0% ACW, (**b**) 10% ACW, and (**c**) 15% ACW; and after impact with 30 Joules: (**d**) 0% ACW, (**e**) 10% ACW, and (**f**) 15% ACW.

However, under an impact energy of 20 Joules, the samples containing ACW exhibited better performance compared to the reference sample (0% ACW), as shown in Figure 16a–c, with the red arrows indicate the impact location. Specifically:

- The samples with 2%, 5%, and 20% ACW presented cracks propagating from the point of impact.
- The sample with 15% ACW showed the formation of both cracks and fissures.
- The sample with 10% ACW exhibited no visible damage.

At the maximum applied energy of 30 Joules, all samples failed due to the impact load. The reference sample (0%) suffered a sudden and complete rupture, as illustrated in Figure 16d–f. Conversely, the samples with 10% ACW, although exhibiting crack propagation, did not progress to total failure, demonstrating a higher capacity for energy dissipation and enhanced toughness under severe impact conditions.

These results suggest that the incorporation of 10% ACW contributes to a synergistic effect, likely due to the presence of fibrous elements within the ACW, which enhances the material’s ability to absorb and dissipate energy under dynamic loading conditions. This behavior indicates a potential for optimized ACW incorporation in applications where greater impact resistance is required, such as floating floors or resilient subfloor systems.

In general, the incorporation of ACW reduced the occurrence of fissures and cracks in the tested samples. However, there is a scarcity of studies specifically addressing this behavior, particularly regarding the incorporation of fibrous materials. According to Mehta and Monteiro [54] and Quinino [55], despite reporting positive outcomes in hard body impact tests on cementitious composites, it remains challenging to accurately quantify the extent of the improvements provided by fiber reinforcement in the matrix. Conversely, studies by Balaguru and Shah [56] and Banthia et al. [57] demonstrated that impact resistance is enhanced through increased energy absorption resulting from the addition of fibers, a finding also corroborated by Bayasi and Zeng [58]. Research by Esaker et al. [59] suggests that impact resistance tends to improve with larger aggregate sizes. However, the influence of aggregate particle size distribution on impact resistance remains unclear and, in some cases, insufficiently explored. These authors emphasize that to enhance impact resistance and energy absorption capacity, the material must exhibit high tensile strength and ductility, thereby highlighting the importance of further investigations into the incorporation of textile fibers in cementitious matrices.

### 3.3. Microscopy Analysis of Cementitious Composites with ACW

**Figure 17:**
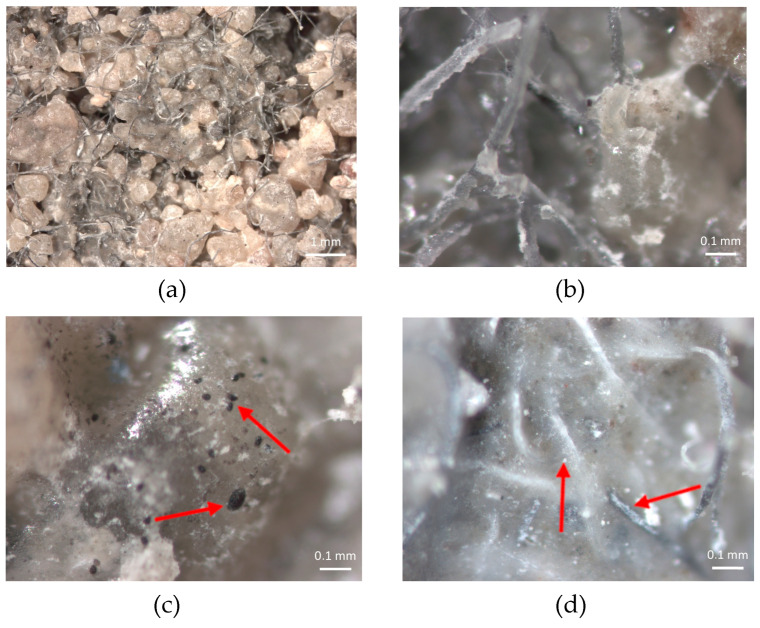
OM images of the sample with 20% ACW: (**a**) mortar/ACW composite (13×), (**b**) ACW fibers (100×), (**c**) ACW particles incorporated into the cement (100×), and (**d**) incorporated fiber (100×).

The microstructure of the mortar/ACW composite was initially analyzed using a stereomicroscope (OM) with magnifications of up to 250×. Figure 17 presents images obtained from fragments of a specimen subjected to compressive strength testing at 28 days, containing 20% ACW incorporated into the cementitious matrix. The images clearly illustrate the successful incorporation of ACW within the mortar, revealing the presence of polymeric fibers and particulate residues embedded in the cement matrix, indicated by the red arrows. Additionally, the morphology highlights the heterogeneous distribution of the ACW, as well as its interaction with the surrounding cement paste.

**Figure 18:**
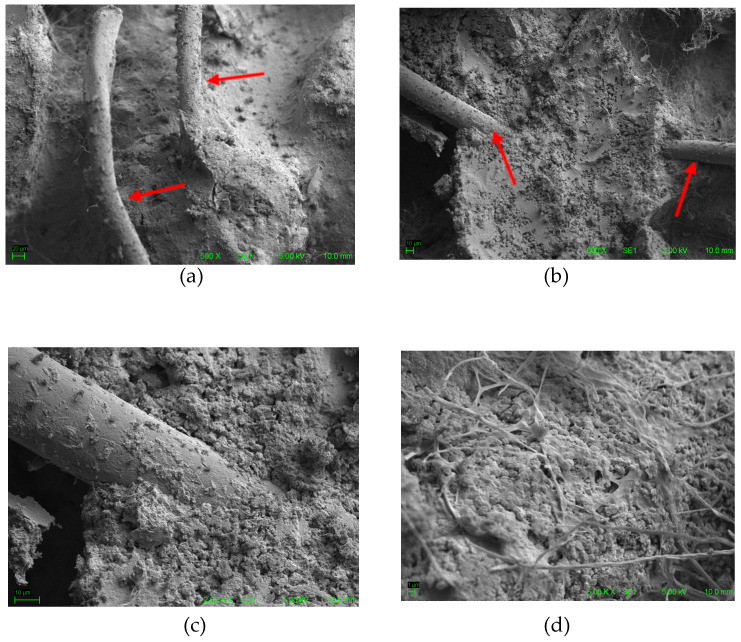
ACW fibers incorporated into mortar observed by SEM at different magnifications: (**a**) 500×, (**b**) 600×, (**c**) 2000×, and (**d**) 6000×.

Subsequently, Scanning Electron Microscopy (SEM) images were obtained (Figure 18), which confirmed the effective incorporation of ACW particles into the cementitious matrix, indicated by the red arrows. The images show no clear distinction at the interface between the carpet residues and the mortar matrix, indicating good adhesion and interaction between the materials. Additionally, it is possible to observe fibrous structures derived from the ACW uniformly embedded within the matrix, demonstrating the morphological integration of the fibers into the cementitious composite.

### 3.4. Dynamic Stiffness of Cementitious Composites Incorporating ACW

The dynamic stiffness tests were conducted by ISO 9052-1 [32] and ISO 12354-2 [42] standards. Table 3 presents the mean values and standard deviations of the resonance frequencies and dynamic stiffness obtained from three specimens tested for each of the five mortar mixtures, along with the calculated impact sound reduction values (∆L). The measured resonance frequencies ranged from 57 to 152 Hz, within the tested acceleration range and the 0–300 Hz frequency sweep, which corresponds to the low-frequency vibration spectrum (<400 Hz).

**Figure 19:**
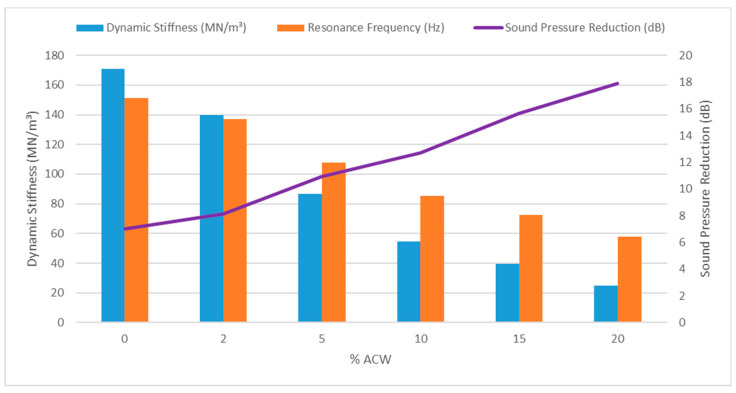
Dynamic Stiffness (MN/m^3^) compared with resonance frequency (Hz) and sound pressure level reduction (dB).

The results indicate that increasing the percentage of ACW incorporated into the composite leads to a decrease in both resonance frequency and dynamic stiffness values (Figure 19). This trend reflects an enhanced capacity of the material to absorb impact-induced vibrations, thereby yielding higher reductions in impact sound pressure level (∆L). Such behavior is consistent with expectations, as the reduction in bulk density—caused by increased porosity from ACW incorporation—facilitates improved vibration damping. Similar findings have been reported in studies involving other types of residues, as seen in studies by Borges et al. [45], Führ et al. [28], and Zuchetto et al. [60].

**Figure 20:**
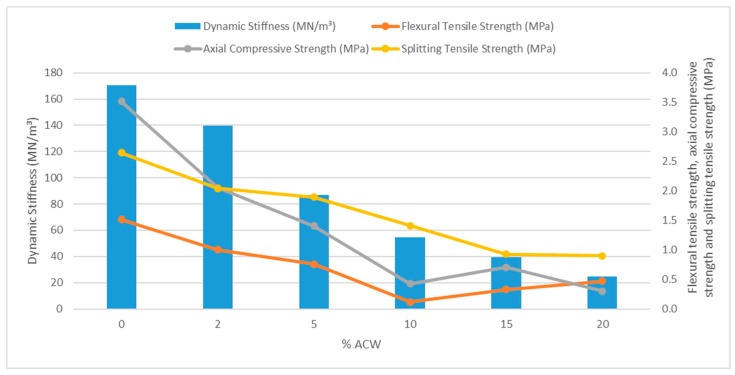
Dynamic stiffness (MN/m^3^) compared to flexural and compressive strength (MPa).

The comparison between the dynamic stiffness and mechanical strength of the samples indicates that lower mechanical strength is associated with lower dynamic stiffness values (Figure 20), particularly in samples with higher ACW content, which exhibit greater potential for reducing impact noise. This behavior demonstrates that, although the incorporation of ACW enhances acoustic performance, it compromises the mechanical properties of the mortars when compared to the reference.

**Figure 21:**
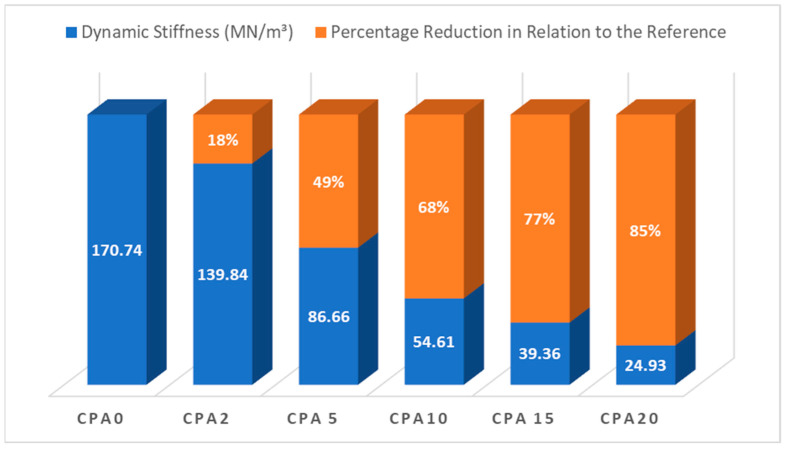
Dynamic stiffness (MN/m^3^) of mortar composites with different ACW contents.

Regarding the incorporation of ACW into the mortar matrix, Figure 21 demonstrates that the dynamic stiffness of the composites is significantly affected. The CPA5 composite, which contains 5% ACW, exhibits a reduction in dynamic stiffness of nearly 50% compared to the reference sample (0% ACW). This trend becomes even more pronounced as higher percentages of ACW are incorporated, resulting in progressively greater reductions in dynamic stiffness. This behavior reinforces the direct influence of the ACW content on the material’s ability to absorb vibrational energy due to the increased porosity and reduced mass density introduced by the fibrous and polymeric nature of the residue.

**Figure 22:**
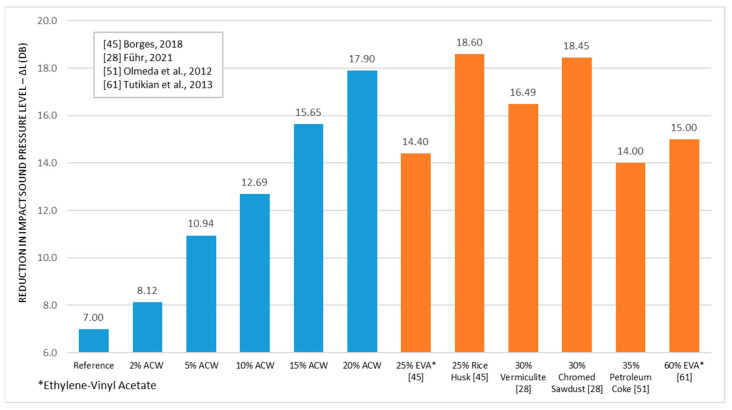
Comparison of acoustic performance with other compounds in the literature [28,45,51,61].

The comparison of the impact sound pressure level reduction (∆L, dB) achieved by the mortar/ACW composites developed in this study with those reported in the literature (Figure 22) reveals comparable results to the works of Borges et al. [45], Führ et al. [28], Olmeda et al. [51], and Tutikian et al. [61], even though these studies employed higher incorporation rates of alternative materials—25%, 30%, 35%, and 60%, respectively. Additionally, the ∆L values obtained in this research are consistent with those found in resilient materials, as evidenced by Neves et al. [62]. These results highlight the acoustic performance potential of ACW-based mortar composites, indicating their technical feasibility as a sustainable alternative to conventional floating floor materials, contributing not only to noise attenuation but also to waste valorization in the construction industry.

Given the results, the identified potential for acoustic enhancement in subfloor systems through ACW incorporation justifies the optimization of mortar formulations. This process may lead to the development of a composite material with excellent acoustic performance, particularly suitable for impact noise insulation in floating floor systems. Additionally, the proposed composite offers environmental benefits as it is based on the reuse of waste materials, aligning with sustainable construction practices. Provided that the balance between mechanical resistance and acoustic efficiency is achieved, this solution proves structurally viable. These findings support the feasibility of using ACW-based mortars as an alternative to conventional materials and encourage further research focused on refining mechanical behavior, durability, and long-term acoustic performance.

## 4. Conclusions

The research goal was to develop and evaluate construction industry composites incorporating waste from ELV carpets, with a focus on maintaining or enhancing performance compared to conventional materials. It was concluded that it is technically feasible to develop cementitious composites incorporating ACW for applications in civil construction, particularly in subfloor systems. However, further studies are necessary to refine the formulation and optimize performance.

The incorporation of ACW into mortars required a higher water/binder (w/b) ratio, primarily due to the high water absorption capacity of the carpet fibers. This condition increased the porosity of the composites, resulting from the evaporation of excess water during the curing process. Consequently, this porosity significantly influenced the reduction of physical and mechanical properties, including mass density, dynamic modulus of elasticity, flexural tensile strength, and compressive strength. Based on the expected behavior of this type of construction material, mechanical strength is clearly the main property to be improved to enable the production and scalability of a product with incorporated ACW.

Based on the results obtained, the following can be concluded:

i. The incorporation of ACW leads to a notable reduction in the mass density of subfloor mortars. Nevertheless, the mixture with 2% ACW maintains density values statistically equivalent to the reference sample without ACW. Additionally, mortars with higher ACW contents, specifically between 10% and 20%, exhibit no significant differences in density among themselves.
ii. The addition of ACW significantly reduced the dynamic modulus of elasticity of the composites. However, no statistically significant differences were observed between the 10% and 15% ACW contents.
iii. The results of the capillarity test did not reveal a consistent behavioral trend, highlighting the need for additional studies to better understand whether higher ACW incorporation facilitates or impairs the development of the pore network governing water transport within the cementitious matrix.
iv. The axial and splitting tensile strength test demonstrated a progressive reduction in mechanical performance as the percentage of ACW incorporated into the cementitious matrix increased.
v. The results of flexural tensile strength showed a similar reduction trend. However, at higher ACW contents, the presence of carpet fibers appears to contribute to a partial recovery or improvement in flexural performance, suggesting a potential reinforcing effect provided by the fibers.
vi. The impact resistance, despite the mortar mix not being optimized for maximum, the results indicate that the incorporation of ACW fibers enhanced the material’s ability to resist crack formation under impact energies of 10 and 20 joules. Furthermore, the sample with 10% ACW exhibited a significant improvement under a 30-joule impact, remaining intact without rupture, highlighting the potential contribution of the fibrous components to energy absorption and damage mitigation.
vii. The dynamic stiffness test demonstrated that all levels of ACW incorporation in the subfloor mortar led to reductions in dynamic stiffness values. This trend suggests that increasing the ACW content leads to a decrease in the dynamic stiffness of the subfloor composite. Such behavior suggests that ACW incorporation in the cementitious matrix can effectively contribute to acoustic insulation against impact noise.
viii. The reductions in impact sound pressure level (∆L), calculated by ISO 12354-2, were consistent with values reported in the literature and exhibited improvements as the percentage of ACW increased, confirming the enhanced acoustic performance of the composites.
ix. The results of microscopy analyses using Optical Microscopy (OM) and Scanning Electron Microscopy (SEM) demonstrated the effective integration of ACW fibers within the mortar matrix, exhibiting good fiber–matrix adhesion and a homogeneous microstructure.
x. The mixture of the four different automotive carpets analyzed in this study resulted in a residue composed of PET, PES, and cotton fibers. This composite residue demonstrated effectiveness in reducing impact noise when incorporated into subfloor mortar.

The development and application of mortar composites incorporating ACW reinforce the principles of the circular economy by transforming ACW into a valuable raw material for another industrial sector. This approach helps mitigate supply challenges faced by the construction industry due to the declining availability of conventional inputs. Moreover, the creation of composites with enhanced acoustic performance offers a sustainable alternative to traditional materials, improving occupant comfort and ensuring compliance with current regulatory standards.

In addition, the practical contributions include the development of a new market opportunity for ELV carpet waste, promoting environmentally sustainable disposal through its incorporation into civil construction composites. This novel application has the potential to mitigate or eliminate disposal costs, enabling the commercialization of this waste as a raw material in the future. Furthermore, it incentivizes vehicle dismantling companies and other environmental service providers to establish and enhance processes related to the collection, processing, and management of this waste stream. These dynamics help foster a more integrated waste recovery chain, encouraging collaboration between the automotive and construction sectors.

Durability tests, such as resistance to abrasion or freeze/thaw cycles, may be prospects for future work with this same material. In real life, technology transfer also depends on the ability of ACW suppliers to adequately process the material and avoid the variability in textures and chemical compositions of different carpets used in automotive manufacturing. Future studies should, therefore, focus on long-term performance assessments of ACW-based composites under real service conditions, as well as on the development of pre-processing techniques that ensure standardization and quality control of the recycled input. Additionally, life cycle assessments (LCAs) and cost–benefit analyses would be valuable to fully understand the environmental and economic implications of scaling this technology.

## Figures and Tables

**Table 1:** Results of tests carried out compiled according to ACW content (%).

Sample	% ACW	Bulk Density (kg/m^3^)		Dynamic Modulus of Elasticity (GPa)		Capillary Water Absorption (g/cm^2^)		Flexural Tensile Strength (MPa)		Axial Compressive Strength (MPa)		Splitting Tensile Strength (MPa)	
		Mean	Std. Dev.	Mean	Std. Dev.	Mean	Max Relative Deviation (%)	Mean	Std. Dev.	Mean	Std. Dev.	Mean	Max Relative Deviation (%)
CP0	0	1687.59	13.22	14.10	0.53	2.00	10.00	1.51	0.10	3.52	0.30	2.64	5.61
CP2	2	1644.09	47.76	11.56	1.16	2.60	9.10	1.00	0.20	2.06	0.30	2.04	5.13
CP5	5	1570.07	17.72	9.37	0.56	3.20	18.80	0.76	0.20	1.40	0.30	1.89	7.66
CP10	10	1291.91	14.96	6.64	0.11	2.00	10.00	0.12	0.00	0.43	0.20	1.41	7.78
CP15	15	1465.52	19.55	6.16	0.76	0.90	7.10	0.33	0.10	0.71	0.20	0.93	7.59
CP20	20	1305.02	22.03	3.98	0.07	2.60	15.40	0.47	0.20	0.30	0.10	0.90	3.92

**Table 2:** Compiled results of Analysis of Variance (ANOVA) for mechanical and physical properties.

Sample	Bulk Density (kg/m^3^)		Dynamic Modulus of Elasticity (GPa)		Capillary Water Absorption (g/cm^2^)		Flexural Tensile Strength (MPa)		Axial Compressive Strength (MPa)		Splitting Tensile Strength (MPa)	
	Mortar	Error	Mortar	Error	Mortar	Error	Mortar	Error	Mortar	Error	Mortar	Error
Sum of Square	429,466	7724	181,141,101	4,506,239	7	2	4	0	45	2	7	0
Degrees of Freedom	5	12	5	10	5	12	5	12	5	30	5	12
Mean Square	85,893	644	9.37	450,624	1	0	1	0	9	0	1	0
F-Test	133.45	-	80.40	-	7.71	-	31.40	-	152.92	-	115.02	-
*p*-Value	0.000000	-	0.000000	-	0.001870	-	0.000002	-	0.000000	-	0.000000	-
Significance	Yes	-	Yes	-	Yes	-	Yes	-	Yes	-	Yes	-

**Table 3:** Resonance frequency fr (Hz), dynamic stiffness (MN/m^3^), and impact sound pressure level reduction ∆L (dB) as a function of % ACW.

Sample	% ACW	Resonance Frequency (Hz)		Dynamic Stiffness (MN/m^3^)		Potential Sound Pressure Level Reduction (dB)
		Mean	Std. Dev.	Mean	Std. Dev.	
CP0	0	151.27	2.18	170.74	4.92	7.00
CP2	2	136.90	1.51	139.84	3.08	8.12
CP5	5	107.77	1.45	86.66	2.35	10.94
CP10	10	85.53	2.25	54.61	2.87	12.69
CP15	15	72.60	2.59	39.36	2.82	15.65
CP20	20	57.70	4.34	24.93	3.82	17.90

## Data Availability

The original contributions presented in this study are included in the article. Further inquiries can be directed to the corresponding author.

## References

[1] Cho S.-J., Jang H.-N., Cho S.-J., Yoon Y.-S., Yoo H.-M. (2023). Material Recycling for Manufacturing Aggregates Using Melting Slag of Automobile Shredder Residues. Materials.

[2] Örücü E., Türkmen B.A. (2022). Evaluating the sustainability of car mat manufacturing. Sustain. Mater. Technol..

[3] Cai Z., Al Faruque M.A., Kiziltas A., Mielewski D., Naebe M. (2021). Sustainable Lightweight Insulation Materials from Tex-tile-Based Waste for the Automobile Industry. Materials.

[4] Widmer R., Du X., Haag O., Restrepo E., Wager P.A. (2015). Scarce metals in conventional passenger vehicles and end-of-life vehicle shredder output. Environ. Sci. Technol..

[5] Wong Y.C., Al-Obaidi K., Mahyuddin N. (2018). Recycling of end-of-life vehicles (ELVs) for building products: Concept of processing framework from automotive to construction industries in Malaysia. J. Clean. Prod..

[6] Arese M., Cavallo B., Ciaccio G., Brunella V. (2025). Characterization of Morphological, Thermal, and Mechanical Performances and UV Ageing Degradation of Post-Consumer Recycled Polypropylene for Automotive Industries. Materials.

[7] Erbs A., Nagalli A., Mymrine V., Carvalho K.Q. (2015). Determinação das propriedades físicas e mecânicas do gesso reciclado proveniente de chapas de gesso acartonado. Cerâmica.

[8] Xu L.Y., Yu J., Huang B.T., Lao J.C., Wu H.L., Jiang X., Xie T.Y., Dai J.G. (2025). Green and low-carbon matrices for Engineered/Strain-Hardening Cementitious Composites (ECC/SHCC): Toward sustainable and resilient infrastructure. J. Clean. Prod..

[9] Coffetti D., Crotti E., Gazzaniga G., Carrara M., Pastore T., Coppola L. (2022). Pathways towards sustainable concrete. Cem. Concr. Res..

[10] Susunaga M.P., Gongora I.A.G., Palmeira E.M. (2025). Evaluation of the Impact of Sustainable Infrastructure on the Perception of the Community Through the Use of Geocells Made of Recycled Tires in an Educational Environment. Sustainability.

[11] Ming Y., Chen P., Li L., Gan G., Pan G. (2021). A Comprehensive Review on the Utilization of Recycled Waste Fibers in Ce-ment-Based Composites. Materials.

[12] Souayfane F., Fardoun F., Biwolfe P. (2016). Phase change materials (PCM) for cooling applications in buildings: A review. Energy Build..

[13] Vasconcelos G., Lourenço P.B., Camões A., Martins A., Cunha S. (2015). Evaluation of the performance of recycled textile fibres in the mechanical behaviour of a gypsum and cork composite material. Cem. Concr. Compos..

[14] Handoko W., Pahlevani F., Emmanuelawati I., Sahajwalla V. (2016). Transforming automotive waste into TiN and TiC ceramics. Mater. Lett..

[15] Go T.F., Wahab D.A., Rahman M.N.A., Ramli R., Azhari C.H. (2011). Disassemblability of end-of-life vehicle: A critical review of evaluation methods. J. Clean. Prod..

[16] Liu P., Farzana R., Rajarao R., Sahajwalla V. (2017). Lightweight expanded aggregates from the mixture of waste automotive plastics and clay. Constr. Build..

[17] Rashad A.M. (2016). A comprehensive overview about recycling rubber as fine aggregate replacement in traditional cementitious materials. Int. J. Sustain. Built Environ..

[18] Ataria R.B., Wang Y.C. (2022). Mechanical Properties and Durability Performance of Recycled Aggregate Concrete Containing Crumb Rubber. Materials.

[19] Khaloo A.R., Esrafili A., Kalani M., Mobini M.H. (2015). Use of polymer fibers recovered from waste car timing belts in high-performance concrete. Constr. Build. Mater..

[20] Eusuf M.A., Al Hasan A. (2013). Study the heat transfer potentiality of a building envelope integrated with elt at foundation. World Appl. Sci. J..

[21] Yu J., Wu Q., Zhao D., Jiao Y. (2025). Influence of Recycled Tire Steel Fiber Content on the Mechanical Properties and Fracture Characteristics of Ultra-High-Performance Concrete. Materials.

[22] Innovation in Textiles (2012). Automotive Fabrics: Expanding Opportunities in the Vehicles of Tomorrow. Technical Textile Market. https://www.innovationintextiles.com/automotive-fabrics-expanding-opportunities-in-the-vehicles-of-tomorrow/.

[23] Atakan R., Sezer S., Karakas H. (2018). Development of nonwoven automotive carpets made of recycled PET fibers with improved abrasion resistance. J. Ind. Text..

[24] Coimbra N.S. (2024). Development of composites for civil construction incorporating automotive carpet waste. Ph.D. Thesis.

[25] Lesiak P., Kisielowska A., Walkowiak K., Wiktorczyk A., Kramek G., Wypych M., Sadkowski L., Zielinski J., Paszkiewicz S., Irska I. (2020). Preparation and characterization of polymer blends based on waste from automotive coverings. Polimery.

[26] (2018). Cimento Portland—Requisitos.

[27] (2022). Agregados—Determinação da Composição Granulométrica—Método de Ensaio.

[28] Führ G., Masuero A.B., Pagnussat D.T., Menna Barreto M.F.F. (2021). Impact sound attenuation of subfloor mortars made with exfoliated vermiculite and chrome sawdust. Appl. Acoust..

[29] Batezini R. (2013). Estudo preliminar de concretos permeáveis como revestimento de pavimento para áreas de veículos leves (Preliminary study on pervious concrete as the surface layer for light traffic areas). Master’s Thesis.

[30] Borges J.G.K. (2015). Análise das propriedades acústicas de contrapisos produzidos com materiais reciclados. Master’s Thesis.

[31] Rossignolo J.A. (2009). Concreto Leve Estrutural: Produção, Propriedades, Microestrutura e Aplicações.

[32] (1989). Acoustics—Determination of Dynamics Stiffness—Part 1: Materials Used Under Floating Floors in Dwellings.

[33] (2005). Argamassa Para Assentamento e Revestimento de Paredes e Tetos—Determinação da Resistência à Tração na Flexão e à Compressão.

[34] (2012). Argamassa de Alta Resistência Mecânica Para Pisos—Determinação da Resistência à Compressão Simples e Tração por Compressão Diametral.

[35] (2021). Standard Practice for General Techniques for Obtaining Infrared Spectra for Qualitative Analysis.

[36] (2015). Standard Test Method for Transition Temperatures and Enthalpies of Fusion and Crystallization of Polymers by Differential Scanning Calorimetry.

[37] (2020). Standard Test Method for Compositional Analysis by Thermogravimetry.

[38] (2005). Argamassa Para Assentamento e Revestimento de Paredes e Tetos—Determinação da Densidade de Massa Aparente no Estado Endurecido.

[39] (2008). Argamassa Para Assentamento e Revestimento de Paredes e Tetos—Determinação do Módulo de Elasticidade Dinâmico Através da Propagação de Onda Ultrassônica.

[40] (2005). Argamassa Para Assentamento e Revestimento de Paredes e Tetos—Determinação da Absorção de Água por Capilaridade e do Coeficiente de Capilaridade.

[41] (2023). Edificações Habitacionais—Desempenho.

[42] (2017). Building Acoustics—Estimation of Acoustic Performance of Buildings from the Performance of Elements—Part 2: Impact Sound Insulation Between Rooms.

[43] Schiavi A., Belli A.P., Russo F., Corallo M. Acoustical and mechanical characterization of an innovative expanded sintered elasticized polystyrene (EPS-E) used as underlayer in floating floors. Proceedings of the 19th International Congress on Acoustics 2007 (ICA 2007).

[44] Brancher L.R., Nunes M.F.O., Grisa A.M.C., Pagnussat D.T., Zeni M. (2016). Acoustic behavior of subfloor lightweight mortars containing micronized poly (Ethylene Vinyl Acetate) (EVA). Materials.

[45] Borges J.K., Pacheco F., Tutikian B., Oliveira M.F. (2018). An experimental study on the use of waste aggregate for acoustic attenuation: EVA and rice husk composites for impact polystyrene expanded reduction. Constr. Build. Mater..

[46] Ahmed H.U., Faraj R.H., Hilal N., Mohammed A.A., Sherwani A.F.H. (2021). Use of recycled fibers in concrete composites: A systematic comprehensive review. Compos. Part B Eng..

[47] Siddique R., Khatib J., Kaur I. (2008). Use of recycled plastic in concrete: A review. Waste Manag..

[48] Rubin A.P. (2015). Argamassas autonivelantes industrializadas para contrapiso: Análise do desempenho físico-mecânico frente às argamassas dosadas em obra. Master’s Thesis.

[49] Fashandi H., Pakravan H.R., Latifi M. (2019). Application of modified carpet waste cuttings for production of eco-efficient lightweight concrete. Constr. Build. Mater..

[50] Asasutjarit C., Hirunlabh J., Khedari J., Charoenvai S., Zeghmati B., Cheul U.S. (2007). Development of coconut coir-based lightweight cement board. Constr. Build. Mater..

[51] Olmeda J., Frías M., Olaya M., Frutos B., Sánchez de Rojas M.I. (2012). Recycling petroleum coke in blended cement mortar to produce lightweight material for Impact Noise Reduction. Cem. Concr. Compos..

[52] Awal A.A., Mohammadhosseini H. (2016). Green concrete production incorporating waste carpet fiber and palm oil fuel ash. J. Clean. Prod..

[53] Tutikian B.F., Zuchetto L.K., Souza R.P., Oliveira M.F. (2017). The use of EVA in mortar floor coverings for impact noise insulation in residential building. Ambient. Constr..

[54] Mehta P.K., Monteiro P.J.M. (2014). Concrete: Microstructure, Properties, and Materials.

[55] Quinino U.C.M. (2015). Investigação experimental das propriedades mecânicas de compósitos de concreto com adições híbridas de fibras. Ph.D. Thesis.

[56] Balaguru P.N., Shah S.P. (1992). Fiber Reinforced Cement Composites.

[57] Banthia N., Yan C., Sakai K. (1998). Impact Resistance of Concrete Plates Reinforced with a Fiber Reinforced Plastic Grid. Mater. J..

[58] Bayasi Z., Zeng J. (1993). Properties of polypropylene fiber reinforced concrete. Mater. J..

[59] Esaker M., Thermou G.E., Neves L. (2023). Impact resistance of concrete and fibre-reinforced concrete: A review. Int. J. Impact Eng..

[60] Zuchetto L.K., Nunes M.F.O., Tutikian B.F. Dynamic stiffness evaluation of floor covering system made out of recycled EVA—Ethylene Vinyl Acetate. Proceedings of the INTER-NOISE 2015—44th International Congress and Exposition on Noise Control Engineering.

[61] Tutikian B.F., Nunes M.F.O., Leal L.C., Marquetto L. (2013). Hormigón ligero con agregado reciclado de EVA para atenuación del ruido de impacto. Mater. Constr..

[62] Neves A., António J., Nossa A. (2008). Resultados experimentais da rigidez dinâmica de materiais usados sob pavimentos flutuantes. Tecniacústica 2008: Conferencias y Comunicaciones de Acústica 2008, Proceedings of the V Congreso Ibérico de Acústica y Tecniacustica 2008.

